# Oceanographers Comb Waters for Genetic Warnings of a Coastal Neurotoxin

**DOI:** 10.1021/acscentsci.5c00558

**Published:** 2025-04-08

**Authors:** Jonathan Feakins

After starting her PhD studies in 2018, Monica Thukral would go for a swim off the coast of San Diego two to three times a week. The regular, physical connection with the waters helped to anchor her in her research at the University of California San Diego’s Scripps Institution of Oceanography.

“I had an understanding of the physical processes: how the temperature and the wave energy change throughout the year,” Thukral recalls. “I was the first to know when an algae bloom was taking place.”

In the laboratory, Thukral transformed that intuition into something more systematic. Using cutting-edge analytical tools, her team in the environmental systems biology lab of Andrew E. Allen—who also teaches at the nearby J. Craig Venter Institute, a genomics research foundation—strove to understand what was happening in the water on a molecular level.

The scientists were working in the wake of a momentous oceanographic event. In 2015, a massive harmful algal bloom (HAB) had subsumed a stretch of Pacific coastline from southern California all the way to Alaska’s Aleutian Islands. It flooded the region’s waters with domoic acid, a neurotoxin so dangerous that it gives sea lions epilepsy; government regulators shuttered some commercial fisheries for months.

**Figure:**
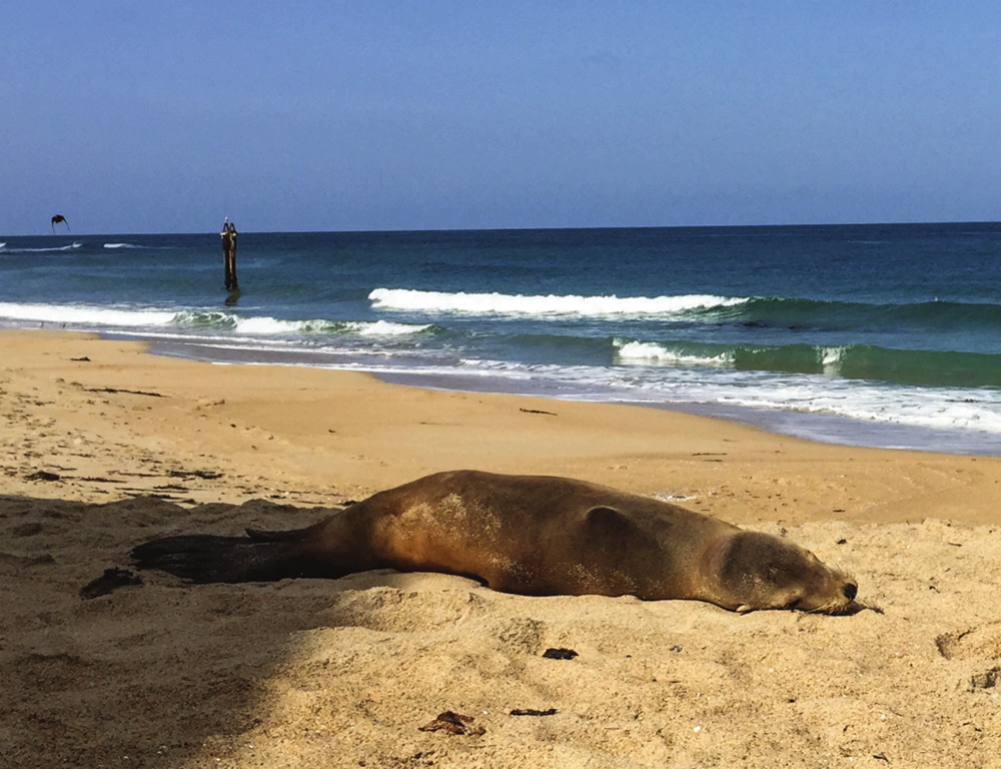
A sea lion shows symptoms of domoic acid poisoning during the harmful algae bloom along the US Pacific Coast in 2015. Credit: Melinda Nakagawa, MBARI

Produced by microalgae—namely, several species of marine diatoms known as *Pseudo-nitzschia*—domoic acid can bioaccumulate in the marine food web, eventually getting consumed by humans, in whom it can cause nausea, cardiac arrhythmia, and a condition called amnesic shellfish poisoning, which can include memory loss and disorientation. Once the acid has accumulated in shellfish tissues past an official threshold of 20 ppm, regulators consider the meat unsafe for human consumption.

The 2015 HAB affected the West Coast more than any other recorded bloom in history, costing an estimated $97 million dollars in damages to the Dungeness crab harvest alone. Domoic acid manifests in all of the world’s upwelling zones—coastal regions where winds push away warm surface water and draw cold, nutrient-rich waters to the surface. The neurotoxin is poised to become even more prevalent as warming oceans disturb the balance of nutrients available.

For years, scientists have been working to understand the mechanisms behind these blooms. “We’ve long held a range of hypotheses in this field for what initiates blooms of this particular organism and its toxin production,” says Clarissa Anderson, one of Thukral’s collaborators and the director of the Southern California Coastal Ocean Observing System (SCCOOS). “We all want to know, What is that magic sauce?”

Now Allen’s interdisciplinary team has figured out a way to potentially anticipate future toxic events. The researchers’ methods center around monitoring the algae-infused waters for environmental DNA (eDNA), material that is minute in concentration but rich in genetic information. In a paper published last year in *Proceedings of the National Academy of Science*, they pulled out all the stops to harvest, isolate, and analyze the genes behind the algal havoc.

## The mechanics of a bloom

Fans of classic cinema may already know domoic acid. In 1961, Alfred Hitchcock found himself inspired by a startling newspaper report about a frenzy of disoriented seabirds terrifying residents of coastal Santa Cruz, California. The director wove elements of that incident into his film *The Birds* two years later. Since then, similar incidents in Monterey Bay have linked addled avians to a diet temporarily infused with domoic acid, a product of Monterey Bay’s occasional HABs.

When stressed, *Pseudo-nitzschia* may release domoic acid for a number of reasons, like to impede the growth of competing plankton or to stave off algae-grazing crustaceans. Also, domoic acid has been found to serve as an iron chelator. The element is critical to photosynthesis, so when it becomes scarce, *Pseudo-nitzschia* will release domoic acid extracellularly, where it can bind to iron in the environment and make it easier for the algae to absorb.

HABs have likely always occurred as part of natural ecological cycles, but modern blooms appear to be becoming more toxic. Anderson says that over the past 20–30 years, deeper waters from the equatorial Pacific have seen rising levels of nitrogen and decreasing levels of silicate. These waters, which came up through the California undercurrent, likely contributed to the size and intense toxicity of 2015’s bloom.

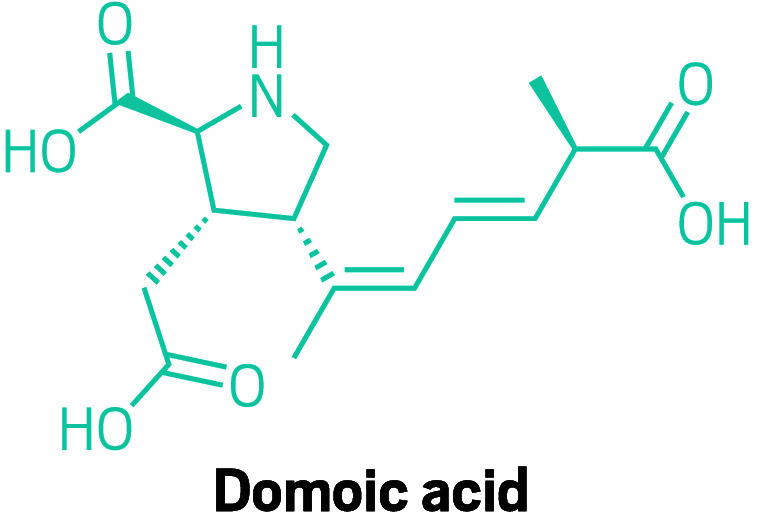



“This massive bloom exhausted silicate before nitrate,” explains John Ryan, a researcher at the Monterey Bay Aquarium Research Institute and coauthor of the paper. Each diatom needs silicate to build its tough cell walls, like a tiny suit of glassy armor. If they run out of silicate, the diatoms cannot divide, but the nitrogen-rich waters continue to fuel their metabolism.

Faced with a silicon shortage threatening their ability to reproduce and an accompanying shortage in essential iron, the nitrogen-charged *Pseudonitszchia* along the Pacific coastline in 2015 took drastic action. The algae started producing as much as 10–20 times their usual amount of iron-scavenging domoic acid. As the compound built up in the *Pseudo-nitzschia*, each morsel of algae that nearby crustaceans ate became “phenomenally toxic,” Ryan says.

In 2018, Thukral’s fellow researcher in Allen’s lab, Patrick Brunson, helped identify DabA, a key enzyme that kick-starts *Pseudo-nitzschia’*s biosynthesis of domoic acid. The paper “was a milestone study,” Allen says. It handed researchers a compass to seek out genes that, like *dabA*, express themselves as a *Pseudonitzchia* bloom takes shape. Now the team wanted to learn what was happening one step before that.

Thukral, Brunson, and the rest of the team figured that because cells express genes before translating them into proteins, they could try to detect genes that *Pseudonitzschia* activated around the same time or even before *dabA*. These could serve as biological alarm bells that a HAB event was imminent.

In the wake of that paper, Brunson found himself the beneficiary of scientific serendipity. Researchers at Moss Landing Marine Laboratories, 650 km up the US Pacific coast from his office at in San Diego, mentioned to Brunson’s team that they happened to have a year’s worth of plankton samples from 2015 sitting quietly in their freezers—including ones chock-full of *Pseudonitzschia* from the bloom.

These were exactly the kind of time-indexed samples that the team needed to take the next step in their research: moving from understanding what caused HABs on a genetic level to identifying the warning signs leading up to a HAB. Things could have gone differently, Allen says. Many discoveries never see the light of day: viable samples languish in freezers, or data gather dust as they wait for robust analysis that never comes, he says.

With the samples in hand, however, the researchers now faced a new challenge. From that slurry of biological material and miscellaneous sea gunk they needed to figure out how to extract and analyze the genetic data lying inside.

## Unveiling genetics

The samples researchers netted during and leading up to the bloom—taken off a municipal wharf in nearby Monterey—provided a veritable goldmine of eDNA, genetic material that organisms shed into their surroundings. It has become an increasingly convenient and nonintrusive tool for researchers to get a picture of ecosystem dynamics.

For example, a 2024 study swabbed hawk beaks and talons for DNA and identified what species of animals the birds preyed upon. And in 2022, biogeochemists extracted the oldest DNA ever from river sediments and reconstructed an ecosystem that had existed in the area millions of years ago.

In addition to analyzing eDNA, Brunson and Thukral gleaned still more information using transcriptomics, a growing subfield of bioinformatics that catalogs the entirety of an organism’s RNA. By seeking out gene transcripts, such as ribosomal RNA, that differ between species, they could chart the evolution of the 2015 bloom—like how *Pseudo-nitzschia australis* had become the dominant diatom by April and had maintained its dominance into autumn.

Aware of the ocean conditions in the days leading up to the bloom—low iron and low silicon concentrations—Brunson and Thukral started to put together the puzzle pieces. Thukral’s efforts uncovered *dabA*’s molecular coconspirator: the gene *sit1*, which helps transport silicon compounds into *Pseudo-nitzschia* cells. She found that when silicon was at its scarcest in early summer 2015, expression of *sit1* skyrocketed as the algal cells tried to eke more silicon out of the water to build their cell walls and divide.

Thukral figured out that when *Pseudo-nitzschia* express both genes at the same time—*dabA* when the algae are starting to synthesize domoic acid and *sit1* as they struggle to divide themselves—it could serve as a “robust predictor” that the *Pseudo-nitzschia* are about to become a toxic powerhouse.

Sorting through and properly analyzing this treasure trove of genetic data—along with data on oceanographic conditions, community composition, and metabolomics—took the team years. “But it becomes pretty beautiful when you’re able to see patterns that are linking them together, and trying to crack the code as to what is taking place in the ocean that we can’t really see with our naked eye,” Thukral says. “All of this data allows us to chip away at that.”

## A bit of notice

The team’s metabolomic investigations fell under the umbrella of a US National Oceanic and Atmospheric Administration (NOAA) research program dedicated to the ecology and oceanography of HABs. Their discoveries from plumbing the depths of environmental genetic data are a step toward something coveted by scientists and fisheries alike: the ability to predict blooms as much as a week before they erupt.

“It’s been exciting, because my field has always been prediction,” says SCCOOS’s Anderson, whose work has involved both remote sensing—such as monitoring HAB formation from space—and developing models that might help predict toxin formation. “But we’re always missing a lot of this fundamental knowledge. I’m really confident that now that we can do some of this molecular forecasting, that we’re a little closer towards a truly mechanistic understanding.”

Vera Trainer, the director of the University of Washington’s Olympic Region Harmful Algal Bloom program, who was not involved with Thukral’s study, will be happy for any help she can get. In the Pacific Northwest of the US, the current warning system has its shortcomings because it requires physically counting phytoplankton cells in water samples and measuring their toxin concentration. By the time high concentrations of toxin can be detected, however, a HAB may already be in progress. “If we had a week’s early warning using these genetic approaches, that would be great,” Trainer says.

She adds that toxic blooms also have an outsized impact on members of the Quinault Indian Nation, on the southwestern corner of Washington’s Olympic Peninsula, who have harvested razor clams for thousands of years. “How do you place a value on the loss of shellfish that you’ve been harvesting for centuries with your family?” Trainer asks. Genetic forecasting using eDNA could allow communities to harvest either before a bloom arrives or in unaffected locations.

“It’s an element ingrained into their lives,” Trainer says. “And when they cannot harvest, it’s damaging not just to their economic well-being, but their cultural well-being.”

**Figure:**
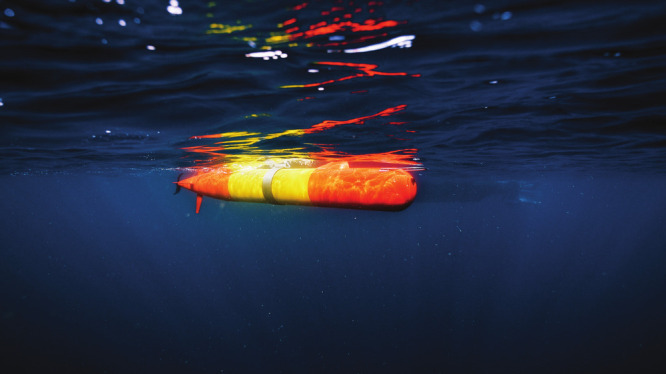
The Monterey Bay Aquarium Research Institute’s autonomous underwater vehicles can prowl water bodies for signs of harmful algal blooms. They take water samples and even do onboard chemical analysis. Credit: MBARI/Monterey Bay Aquarium.

## Next steps

One of the relative weaknesses of the team’s data had nothing to do with their abilities. Their limitation lay in not having enough samples from the beating heart—the initiation sites—of the HABs.

“Where we really want to go next is more directed bloom sampling,” Brunson explains. For samples like those taken by the Moss Landing researchers at a local wharf, “you’re getting a unique environment that might not be precisely like the epicenter of the bloom.” As such, the gene expressions of the HAB that occurred on Moss Landing’s doorstep still need to be confirmed against those of other regions.

Brunson and Thukral’s fellow researchers back at the Monterey Bay Aquarium Research Institute have spent years developing new ocean technology that could provide these genetic samples with robotic precision. Their autonomous underwater vehicles (AUVs) can zoom forth into the ocean depths at a moment’s notice and vacuum up eDNA like an Atlantean Roomba.

The AUV is outfitted with sensors that can continuously hunt for environmental markers so it can pilot itself into the thick of a HAB. This vehicle can then take “sips” of the surrounding water and store what it finds in one of dozens of filters. Researchers then have the choice to bring those samples ashore for hands-on analysis or have the AUV’s onboard systems get a quick look at their molecular components. Ryan says the AUV is “basically a laboratory in a can” that can lyse cells and take a look at their contents using a light-based analysis technique called surface plasmon resonance that can detect domoic acid.

“Once you prepare that vehicle with all the reagents inside, you need to wait for the right moment,” Ryan says. “Maybe it’s a mortality event: you see animals dying from domoic acid poisoning. You then remotely send your AUV on its mission to 1) map the phytoplankton distributions, and 2) report back real-time detection information.”

NOAA does, in fact, already have a fleet of ocean gliders monitoring the oceans, “but most of them aren’t out sniffing HABs,” Anderson explains.

SCCOOS has also stationed along the California coastline a fleet of a dozen robotic microscopes, dubbed Imaging FlowCytobots, that can photograph and identify each phytoplankton in 15 mL of water every hour.

Another set of approaches that could one day complement monitoring and data collection are prevention, control, and mitigation. In other words, scientists could use what they know to try to proactively intervene in natural processes to stop HABs from proliferating.

That line of thinking raises pragmatic and philosophical questions: Will advances in molecular forecasting lead to techniques that can circumvent HAB formation, and can the consequences of this sort of geoengineering be known?

**Figure:**
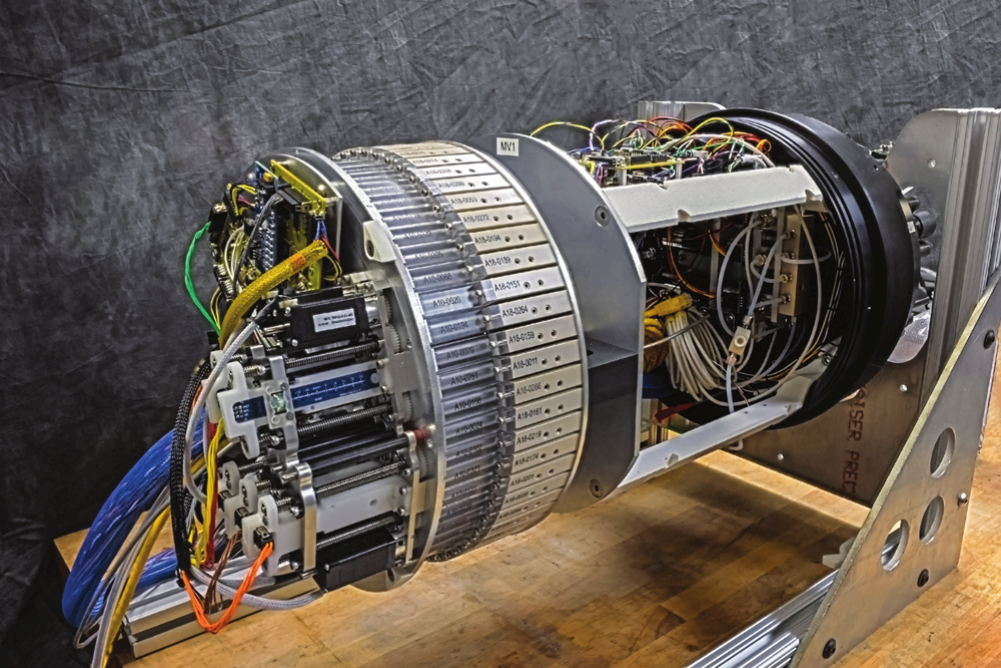
Inside the hull of Monterey Bay Aquarium Research Institute’s autonomous underwater vehicles is an onboard system that can retain the samples for hands-on testing in the lab. Credit: Todd Walsh. © 2018 MBARI.

“Once that horse has left the stable, it’s left the stable,” Anderson admits. In systems the size of the world’s oceans, preventive actions would require intervention on truly massive scales. “They’re much more effective in closed, freshwater systems, and there’s a ton of work being done in that field—with a lot of industry buy-in because they can make all kinds of chemicals that can effectively deal with this. But I do believe that [prevention, control, and mitigation] aspirations in the marine environment are a lot trickier.”

Going forward, however, the transcriptomic techniques that Brunson and Thukral used will also continue to bloom. The sheer amount of biological data circulating through the environment provides more material than could be analyzed by any one laboratory or in any one lifetime.

“That’s one of the amazing things about such a rich data set like this: there’s always so much to explore and learn,” Thukral says.

## Jonathan Feakins is a freelance contributor to

Chemical & Engineering News, *the independent news outlet of the American Chemical Society.*

